# Optimization study of plasmonic cell fusion

**DOI:** 10.1038/s41598-022-11168-x

**Published:** 2022-05-03

**Authors:** Julia Belansky, Dvir Yelin

**Affiliations:** 1 Russel Berrie Nanotechnology Institute, Technion, 32000 Haifa, Israel; 2 Faculty of Biomedical Engineering, Technion, 32000 Haifa, Israel

**Keywords:** Other photonics, Nanoscale biophysics, Cancer models

## Abstract

Artificial cell fusion often serves as a valuable tool for studying different applications in biology and medicine, including natural development, immune response, cancer metastasis and production of therapeutic molecules. Plasmonic cell fusion, a technique that uses specific cell labeling by gold nanoparticles and resonant femtosecond pulse irradiation for fusing neighboring cells, has been demonstrated useful for such applications, allowing high cell specificity and an overall low toxicity. Despite these advantages, the numerous experimental factors contributing to plasmonic fusion have often led to subpar fusion efficiencies, requiring repeated experiments and extensive calibration protocols for achieving optimal results. In this work we present a study that aims to improve the overall performance of plasmonic cell fusion in terms of fusion efficiency and cell viability. By varying the pulse fluence, nanoparticle concentration, incubation times, and culture handling protocols, we demonstrate up to 100% fusion of malignant epithelial cells across the entire irradiated area of the culture. We also show that some of the smaller cells may stay viable for up to several days. The results would allow plasmonic fusion to play a key role in numerous studies and applications that require specific, high-efficiency cell–cell fusion.

## Introduction

Triggering fusion between cells is a valuable tool for various applications in medicine and biotechnology, including the production of monoclonal antibodies^1,2^, in vitro fertilization^3^, cancer immunotherapy^4,5^, gene therapy^6^, improving cerebellar motor function^7^, and the regeneration of skeletal muscle^8^, liver^9^, neurones^10^, and intestinal tissue^11^. For basic research, artificial cell fusion may be used for studying the mechanisms of cancer metastasis^12–17^ and embryo development^18^, and recently for studying the actin networks that are formed during fusion^19^. Despite the wide variety of these applications, artificial cell–cell fusion is often generated using only a relatively small selection of methods for triggering the fusion process. By adding polyethylene glycol (PEG) to the culture medium, the cell membranes tend to fuse together and form multinucleated cells^20^. While this method is relatively simple and straightforward, PEG-induced fusion is often ineffective for cells in suspension^21^, may be cytotoxic at high concentrations^21,22^, and affects all cell types within the culture. In a recent paper by Yoshihara et al. (2020), the PEG-induced fusion efficiency was improved by modifying the cell membranes using oligopeptides that were covalently conjugated to the PEG. As a result, they have reported an efficiency increase from 8.4% to 64% for homogeneous cell fusion, and from zero to 18% for heterogeneous fusion^23^. A more specific method for cell fusion uses transfection by viruses that form a cytoplasmic bridge between cells or induce cell swelling^24,25^. This method was shown effective using the Sendai virus (HVJ) with cells presenting the HVJ receptor^26^; however, viral transfection is often less effective as a general-purpose technique, has relatively low efficiency^21^ and requires high biosafety standards. A more efficient approach for cell fusion has been demonstrated using strong electric fields that could be applied locally for disrupting the cell membranes and promoting fusion^27,28^. Alternating electric fields could also be used to bring cells together^29,30^ for higher fusion efficiency. Recently, He et al. (2019) have compared between PEG-induced fusion and electrofusion in a single integrated microfluidic device, allowing two cells to be brought into contact with 80% success rate, followed by electrofusion efficiency of 26% and PEG-induced efficiency of 21%^31^.

Thanks to the advance of laser technology in recent decades, tightly focused laser beams have been shown useful for fusing selected cells using UV lasers^32^ or near-infrared femtosecond lasers^33,34^. The intense optical fields generated by the lasers lead to localized optomechanical interactions that allow high spatial and temporal accuracies, and with specific laser tweezer configurations, the laser may also be used for bringing together selected cells^32^. Hot-particle-mediated fusion^35^ was recently demonstrated using gold nanoparticles trapped between the optically trapped cells. While the efficiency of this method was not reported, Calcein AM assay has shown that the cells have remained viable for up to 4 hours^35^. This approach is particularly suitable for specific applications such as *in-vitro* fertilization^36^, which require high efficiency when fusing together only a few cells.

In general, the main challenge of all current techniques for generating cell fusion is the apparent tradeoff between fusion efficiency and selectivity, i.e., most methods are not suitable for applications that require specific fusion within a heterogeneous cell population. Recently, our research group has demonstrated a new technique, termed plasmonic cell fusion, for inducing widespread cell fusion using femtosecond laser pulses and gold nanoparticles that are specifically attached to the target cells. Following irradiation of the cell culture by a series of resonant pulses^37^, the local light-particle interactions induce multiphoton ionization^38^ and cavitation bubbles around individual particles^39–41^, causing local membrane disruptions that trigger fusion between two or more neighboring cells^37^. Plasmonic cell fusion was demonstrated promising for stimulating an immune response by fusing malignant and immune system cells^42^ and for creating large actin networks in fused cancer cells^19^. The main advantage of plasmonic fusion is its ability to induce a widespread fusion across the entire irradiated cell culture of tissue, yet only at specific locations determined by the nanoparticles, which can target only specific populations of cells.

Yet, despite the numerous parameters that could be controlled to achieve the desired effect, most of our plasmonic fusion experiments had suffered from varying and often low fusion efficiencies that were strongly dependent on the exact experimental parameters. In this work, we study the effect of some of the key parameters that contribute to the plasmonic fusion of cancer cells, including particle synthesis protocols, particle delivery and laser intensity, and identify an experimental parameter space that allows for nearly 100% cell fusion, as well as for generating individual cells that remain viable for more than 24 h, and also appear to have higher motility levels^43^ compared to the unfused cells.

## Methods

### Cell culture

MDA-MB-468 epithelial cells (American Type Culture Collection) were grown at 37 °C and 5% CO_2_ in a DMEM medium (Invitrogen). The medium was supplemented with 2 mM glutamine and 5 nM sodium pyruvate in addition to 10% heat-inactivated fetal bovine serum. Cell cultures were seeded in an eight-well glass-bottom chamber slide.

### Nanoparticle preparation

To prepare monodispersed gold suspension of nanospheres of 20 nm diameter, gold chloride was reduced with sodium citrate in an aqueous solution^44^. Gold nanoparticles were stabilized by OPSS-PEG2000-NHS (Creative PEGWorks) which was also used to covalently couple anti-EGFR monoclonal antibodies (Thermo Scientific, clone designation EGFR.1) to the particles^45^. Particle size was determined using absorption spectroscopy, and particle attachment to the cells was occasionally verified using 2-photon microscopy.

### Fluorescence labeling

Staining for necrosis was performed using 1 µg/ml Propidium iodide (Sigma). A viability endpoint assay was conducted using 1 µM Calcein AM (Sigma) staining together with 1 µM Propidium iodide (Sigma). The cells were incubated for 10 min at 37 °C and 5% CO_2_ with medium-Calcein AM-Propidium iodide solution prior to imaging.

### Cell targeting by gold nanoparticles

The MDA-MB-468 cells (2 × 10^5^–3 × 10^5^ cells/ml) were incubated for 1–4 h at 37 °C and 5% CO_2_ with different concentrations of anti-EGFR-coated gold nanospheres. Following incubation, cells were washed off unbound nanoparticles (three medium washes) prior to laser irradiation. Control experiments that were conducted in parallel included the irradiation of cell cultures without nanoparticles.

### Laser irradiation

A beam from a Ti: sapphire amplifier (Spitfire Pro XP and MaiTai, Newport Corp) was wavelength-tuned to 550 nm using an optical parametric amplifier (Topas-C, Spectra-Physics), for matching the plasmonic resonance of the gold nanospheres. Pulse duration was 45 fs at a 1 kHz repetition rate. Cells were irradiated within 8-well chamber slides (Lab-Tek II, Thermo Scientific) which were placed within a microscope incubator (Okolab Inc.) at controlled temperature and CO_2_ concentration. The irradiation pattern was a 30 × 30 array of 330 μm diameter spots, covering the total area of 1 cm^2^. Multiple pulse irradiations per spot were achieved by scanning the Gaussian beam at lower rates so that each point was irradiated by several consequent overlapping spots.

### Phase-contrast time-lapse imaging

Phase contrast time-lapse imaging was conducted using a Ti-E Nikon microscope. The objective lens was 10x, NA = 0.3. Time-lapse imaging was performed using NIS-Elements Advanced Research (Nikon) software. A single frame was captured every 5 min during the first 10 h, followed by a frame rate of one frame per 15 min during the rest of the experiment.

### Data analysis

**Figure 1:**
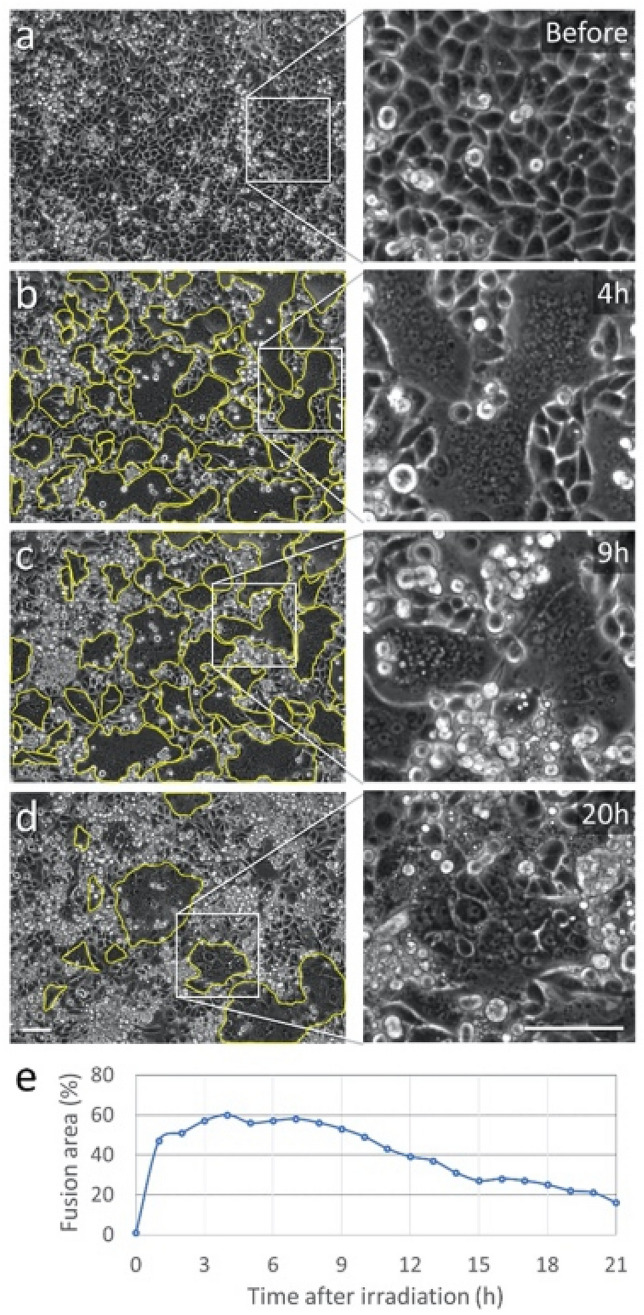
Widespread plasmonic fusion in MDA-MB-468 cancer cells. (**a**) Phase-contrast image of the cell culture before irradiation. Cell membrane is visible as rounded bright curves in the 4× magnified region (right panel), showing a fully confluent culture with an average cell diameter of approximately 10 µm. Dead cells or cells detached from the glass substrate are visible as brighter and smaller circles. (**b**) 4 h after irradiation the relative fusion area (marked by yellow curves) have reached a maximum of 60%. (**c**) The culture has remained stable for up to 9 h after irradiation. (**d**) 20 h after irradiation, most of the multinucleated cells underwent necrosis, where only a few survived, covering approximately 21% of the field of view. Scale bars represent 50 µm. (**e**) Total culture area covered by fused cells for different times after irradiation.

Images were initially processed using ImageJ software, adjusting the color, brightness, contrast and gamma. The fused cells were first identified by the disappearance of the membranes (visible as bright rounded curves) from the phase contrast images. The relative fusion area was calculated using manual segmentation of these cells (yellow curves in Fig. 1). The relative fusion area was then calculated by dividing the total area (number of pixels) of the traced fused cell by the total number of pixels within the field of view. Necrosis measurement was conducted using automatic counting of isolated spots of PI staining. The standard deviation for the relative fusion area was calculated assuming a binomial distribution.

## Results

### Fusion area analysis

Prior to laser irradiation, plasmonic cell fusion requires the attachment of a sufficient number of particles to the cell membranes, whereas excessive particle density may be toxic to the targeted cells and lead to rapid cell necrosis following laser irradiation^46^. Initial calibration experiments with the MDA-MB-468 breast cancer cells and irradiation parameters similar to those reported earlier^37,42,46^ have shown (Supplementary Fig. 1) that fusion area was highly dependent on the initial particle concentration within the incubation medium, as well as on the incubation times, where incubation duration of approximately 3 h was found to be optimal for particle concentrations above 6 × 10^10^ particles/ml. The 3-h incubation time was found to be optimal for practically all particle concentrations, including concentrations higher than 6 × 10^10^ ml^−1^.

In order to observe and quantify the progression of plasmonic fusion within a fully confluent, monolayered cell culture, adenocarcinoma MDA-MB-468 cells were incubated for 3 h in a medium containing 15 × 10^10^ EGFR-specific gold nanoparticles per ml (Fig. 1a). After washing off the unbound particles from the culture medium, the cells were irradiated by a series of 15 pulses with 25 mJ/cm^2^ per pulse, while keeping all other irradiation parameters similar to those used in our previous studies^19,37^, i.e., 45 fs pulse duration, 1 kHz pulse repetition rate, and a central wavelength of 550 nm for matching the plasmonic resonance of the 20-nm-diameter gold nanospheres. After irradiation, the cells were imaged continuously over the course of several hours, resulting in the time-lapse image series shown in Video 1. While the physical effect of the membrane rupturing was practically instantaneous, the bright boundaries between the individual cells have begun to disappear in numerous locations only several minutes after irradiation, leaving darker regions of large multinucleated cells. Four hours after irradiation, the bright boundaries between the cells were almost completely disappeared, leaving several giant cells comprised of dozens of nuclei (enclosed by yellow curves in Fig. 1b) that cover approximately 60% of the field of view. Here and throughout this work, we will refer to this figure, i.e., the relative area covered by fused cells in a fully-confluent culture, as a measure of the efficiency of our method. Most of the resulting multinucleated cells have remained unchanged for up to 9 h after irradiation (Fig. 1c), yet numerous dying cells were detaching from the glass surface and became visible as smaller bright circles. After 9 h, the giant multinucleated cells were gradually losing their structural integrity and die (Fig. 1d). At that stage, most of the remaining multinucleated cells were relatively small and contained only several (3–5) individual nuclei. The relative culture area covered by multinucleated cells is plotted in Fig. 1e as a function of time after irradiation, showing the rapid increase in fusion area up to 1 h after irradiation, a plateau region (1–9 h) in which the multinucleated cells remain relatively stable, and a gradual decrease in fusion area corresponding to the dying fused cells (9–21 h).

Similar experiments with cell cultures that were not labeled by the nanoparticles have shown no fusion events in all of the cultures. In contrast, the cell damage levels visualized by PI staining for necrosis (data not shown), were similar to those in the nanoparticle labeled cultures, indicating that any residual damage to the irradiated cells was caused only by the laser light itself and not by the presence of the nanoparticles.

### Particle concentration and laser power

**Figure 2:**
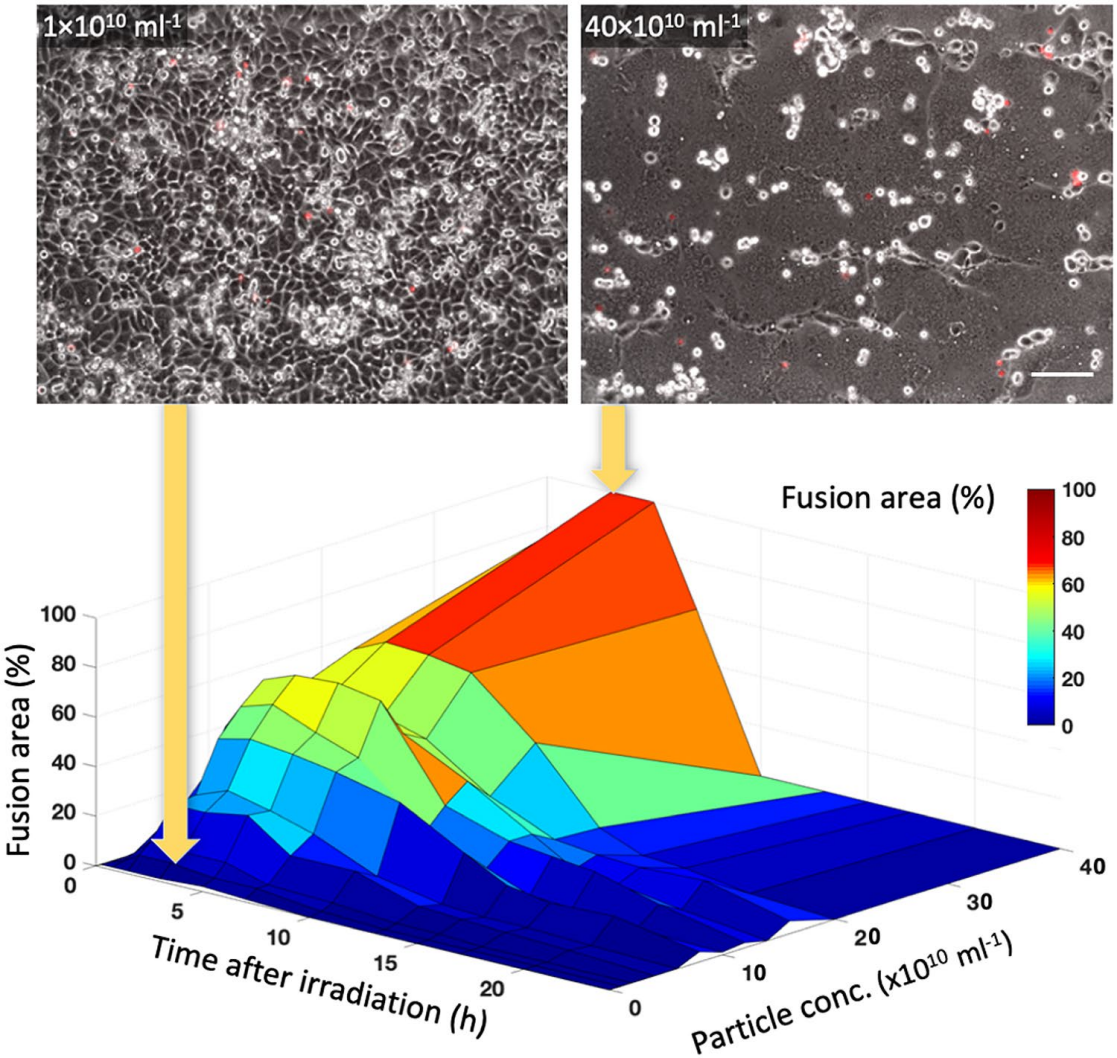
Progression of the relative fusion area for different particle concentrations. The top panels show the cell cultures for the two most extreme concentrations that were tested, 3 h after irradiation. Red stained cells correspond to necrotic cells. Scale bar represents 50 µm.

The effect of particle concentration within the culture medium was studied by irradiating fully-confluent cell cultures at variable particle concentrations and a fixed laser parameter set (15 pulses, 1 kHz, 45 fs, and 35 mJ/cm^2^ per pulse) while measuring the relative fusion area at different times after irradiation (Fig. 2). Evidently, higher particle concentrations have led to higher fusion areas, until 100% of the culture area was covered by what seemed to be a single giant cell comprised of hundreds of nuclei (at 40 × 10^10^ ml^−1^, 3 h after irradiation). Nevertheless, the giant cells formed within the cultures with the higher particle concentrations did not survive for more than 6 h after irradiation, whereas cell death was less significant at particles concentrations below 10 × 10^10^ ml^−1^, most likely due to the typically smaller size of the fused cells which comprised of only a few nuclei.

Clearly, the main challenge in most of the applications that require artificial cell fusion is to maintain the viability of the fusion products for long durations. The experiments summarized in Fig. 2 have shown that some of the fused cells have survived for more than 20 h after irradiation, mainly for the moderate concentrations between 5 × 10^10^ and 15 × 10^10^ ml^−1^.

**Figure 3:**
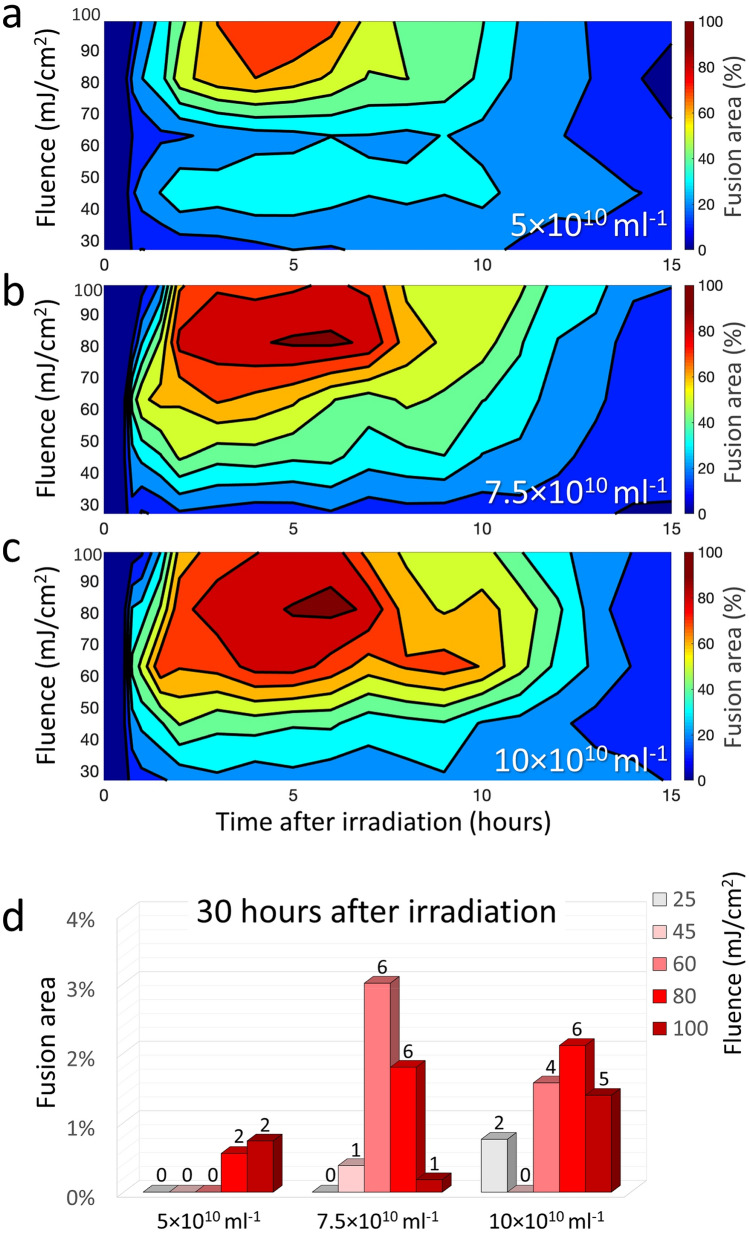
Relative fusion efficiency as function of fluence levels and times after irradiation for three nanoparticle concentrations of (**a**) 5 × 10^10^ ml^−1^, (**b**) 7.5 × 10^10^ ml^−1^, and (**c**) 10 × 10^10^ ml^−1^. Higher particle concentrations have resulted in larger areas of fused cells, even for moderate fluence levels. (**d**) Percent of viable fused cells 30 h after irradiation by the different fluence levels. The number above each bar represents the total number of multinucleated cells in the respective culture.

In order to improve cell viability and to extend the time in which these remain alive, additional experiments were performed for three particle concentrations in this range (5 × 10^10^, 7.5 × 10^10^, and 10 × 10^10^ ml^−1^) while varying the pulse fluence levels between 25 and 100 mJ/cm^2^ (Fig. 3). At the lower 5 × 10^10^ ml^−1^ concentration (Fig. 3a), the total fusion area was gradually increasing with pulse fluence, reaching a maximum of 80% fusion for 100 mJ/cm^2^. At 7.5 × 10^10^ ml^−1^ (Fig. 3b), the fusion was more efficient for all fluence levels, increasing almost linearly with pulse fluence until a maximum fusion efficiency of 92% was obtained for 80 mJ/cm^2^. Further increase in particle concentration to 10 × 10^10^ ml^−1^ has led to the additional expansion of the high-efficiency parameter space down to fluence levels below 70 mJ/cm^2^ (Fig. 3c). For all concentrations, fluence lower than 25 mJ/cm^2^ did not yield any notable fusion, while fluence levels higher than 110 mJ/cm^2^ have always resulted in excessive cell damage and essentially no fusion.

At times longer than 15 h after irradiation, only several relatively small multinucleated cells have remained viable within the culture, covering less than 3% of the culture area. Shown in Fig. 3d is the relative fusion area 30 h after irradiation for the three particle concentrations and the various fluence levels that were tested. A maximum of six multinucleated cells have survived for particle concentration and pulse fluence of 7.5 × 10^10^ ml^−1^ and 60 mJ/cm^2^, respectively, covering approximately 3% of the culture area.

### Cell viability and phenotype

As seen in Figs. 1, 2 and 3, after the fusion area has reached its maximum, the large fused cells were rapidly losing their viability and die, where the largest cells often die faster. While numerous factors may contribute to viability loss in these cells, including the high cell confluence, genetic instability and generation of ROS^46^, the large size of the new multinucleated cells seemed to play the major role in cell death, as evident by the rapid detachment of the cells from the glass substrate.

In order to increase the number of fused cells and improve their overall viability, the culture medium was replaced one hour after irradiation for removing some of the dead cells and reduce the levels of ROS. The large space cleared by the dying cells may allow the surviving smaller cells to move freely across the culture, and the medium replacement would result in improved cell viability. Furthermore, the smaller cells would now have sufficient space to develop components essential for cell motion such as lamellipodia, filopodia and membrane ruffles, and their motility could serve as an indication for their viability. The medium replacement was then repeated every 12 h during the time-lapse imaging of the cultures.

**Figure 4:**
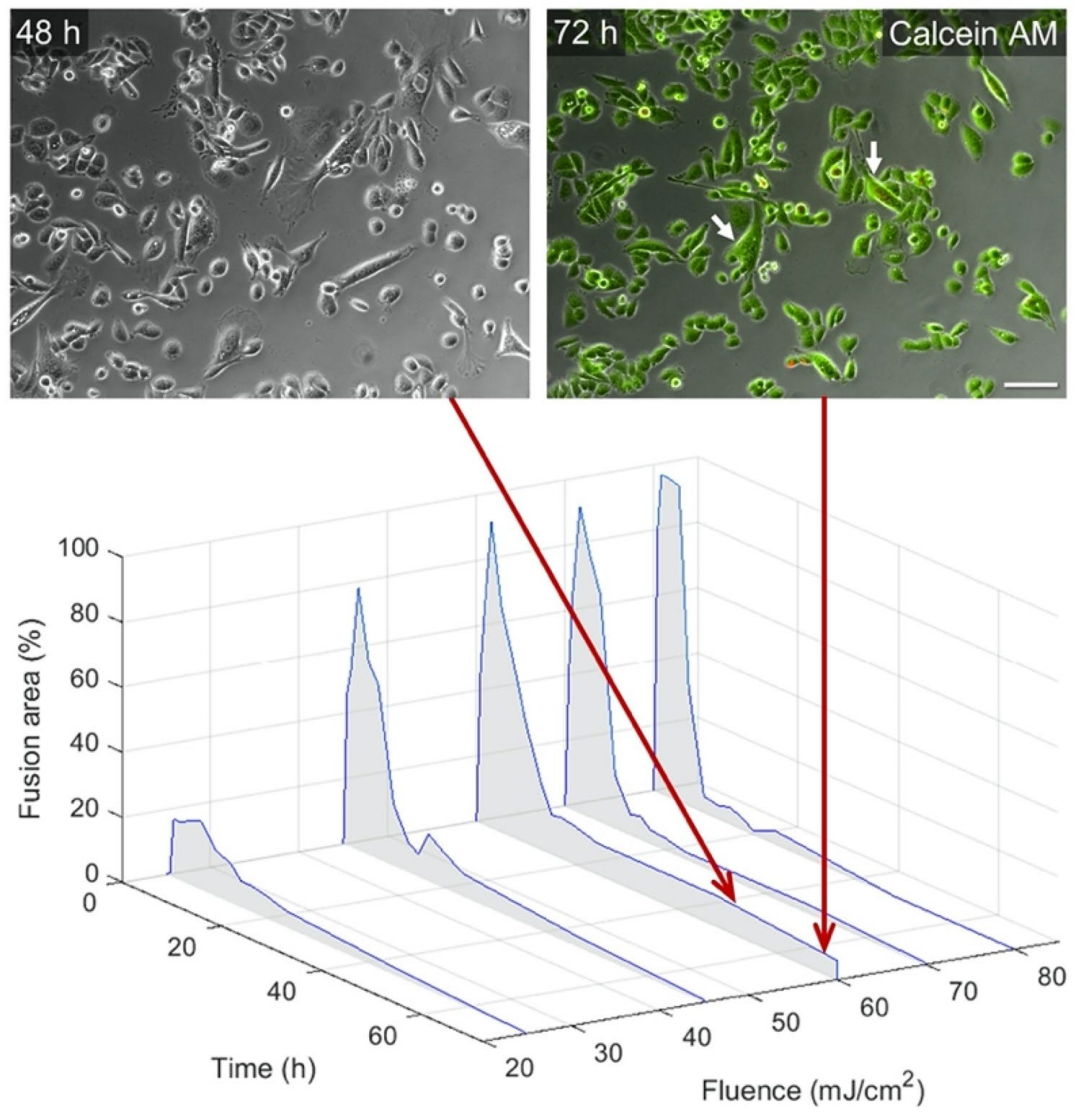
Survival of the fused cells 72 h after irradiation. A few multinucleated fused cells comprised of 2–5 nuclei had remained viable after 60 mJ/cm^2^ pulse fluence, as evident by the Calcein AM green fluorescence and the lack of Propidium Iodide (red) staining. Scale bar represents 50 µm.

Using the new protocol for maintaining cell viability, the fusion experiments were repeated for particle concentration of 7.5 × 10^10^ ml^−1^ under different pulse fluence levels (Fig. 4). During the first 15 h after irradiation (with a single medium replacement), all cultures have shown widespread cell fusion, similar to the previous experiments without medium replacements. During the following hours and up to 72 h after irradiation, most of the fused cells have died in most of the cultures, except for the cultures that were irradiated by 60 mJ/cm^2^, which maintained several multinucleated fused cells that covered up to 8% of the culture area. A Calcein AM staining was used to confirm cell viability in the culture 72 h after irradiation (Fig. 4, top-right panel, arrows indicate viable fused cells). Note that while the total fusion area is much smaller now, the number of the multinucleated cells (3 cells) is only somewhat smaller than the number of giant cells that covered most of the culture (7 cells).

**Figure 5:**
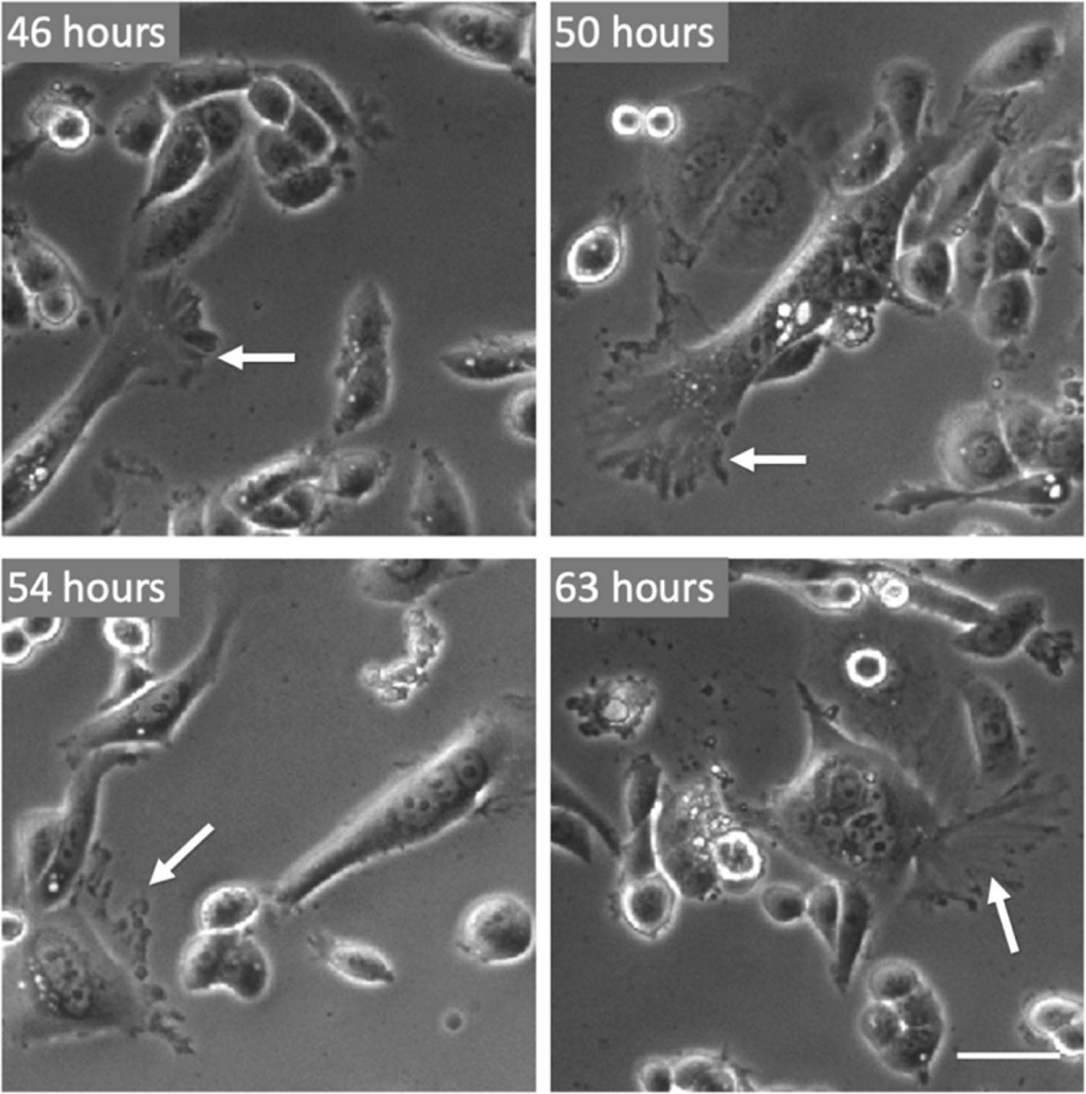
Four examples for the typical phenotype of the small multinucleated MDA-MB-468 cells. The fused cells develop large lamellipodia and filopodia (white arrows) that change continuously over time. Scale bar represents 10 µm.

The widespread death of the giant cells and the subsequent culture washing had resulted in lower cell confluency which allowed additional space for cell movement. In contrast to the unfused cells within the culture, which appear small and relatively motionless, the fused cells appeared larger (Fig. 5), contained several (3–6) nuclei, and showed well-developed lamellipodia, filopodia and membrane ruffles (Fig. 5, arrows). These cells were considerably more mobile than the individual cells within the culture (Supplementary movie 2), dynamically changing their contact area with the glass substrate by extending and contracting their plasma membranes in the cleared regions around them. Such motion would be essential for allowing cell motility, phagocytosis and the development of cell adhesion^47^.

## Discussion

The first observation of plasmonic fusion^37^ was during a study on cancer cell destruction, where widespread fusion was discovered at medium-level irradiation (4 laser pulses), between cell apoptosis (1 pulse) and cell necrosis (16 pulses). Obtaining better control over this unique fusion process required us to tune the initial parameters for improving efficiency and maintaining cell viability for long durations; however, we have found that plasmonic cell fusion was extremely sensitive to almost all parameters of the experimental protocol.

We have started by first determining basic parameters that are essential for obtaining fusion. Gold particles were chosen to allow strong resonances at the visible-NIR range with optimal biocompatibility; particle shapes was chosen as spheres to allow shape stability under intense irradiation that can melt the particles; particle diameter was set to 20 nm, mainly because of the consistency and reliability of the particle preparation protocols and for reducing aggregation^48,49^; antibody concentration was determined according the capacity of the particles, were in general more antibody have led to better results; and finally, an incubation duration of 3 h was determined for saturating the process of particle attachment to the cells’ membranes. Cell confluency in the culture, i.e., the relative substrate area covered by the cell, was chosen as 100%, mainly for achieving comparable results between different experiments. Obviously, lower confluency would result in less cell fusion, and other phenomena may become more dominant in such settings, including optoporation of the plasma membranes^50^.

Some irradiation parameters were also kept constant for maintaining fusion, including the 550 nm wavelength for matching the plasmonic resonance of the particles, 1 kHz pulse repetition rate which is the highest rate achieved by our amplifier system, and a number of 15 pulses per irradiated spot, which under these parameters provided the most efficient fusion.

In the current work we attempted to optimize the experimental parameters that showed more flexibility in tuning the fusion efficiency. After studying the temporal evolution of the irradiated cultures, from the time of irradiation until the cells die (Fig. 1), we have calibrated the particle concentration in conjunction with the irradiation fluence, which essentially determine the amount of energy delivered to the cells for destabilizing its plasma membrane. The result of a nearly 100% fusion was achieved under these specific conditions, and may represent the optimal conditions for generating giant cells that comprise of hundreds of nuclei. While such cells could be used for studying the cells’ cytoskeleton^19^, much smaller cells are clearly needed for practically any other application. Moreover, cell viability may play a more important role for many applications that require healthy, long-lived cellular entities. One obvious solution for this problem is to irradiate a culture that is not fully confluent, or alternatively to use a parameter set that induces less efficient fusion. In general, the resulting smaller, multinucleated cells have lived much longer than the giant cells. By replacing the culture medium and removing the dead cells, we have managed to maintain the cells alive and viable (as confirmed by Calcein AM assay) even after 72 h. Additional experiments may be required to further extend the cells lifetime, depending of course on the specific application that is planned for these cells.

In a broader context, plasmonic fusion offers a unique alternative to the commonly used artificial cell fusion methods. Depending on the application, it allows to induce widespread fusion across an entire cell culture or tissue, yet with cell specificity that can be determined by the selective attachment of the nanoparticles. Still, the main limitation of plasmonic cell fusion is its overall complexity, as it requires specific nanoparticles, careful cell culture preparation, and a dedicated laser system that can provide the intense, resonance femtosecond pulses. Other limitations may include the generation of ROS^46^, the gold nanoparticles that remain attached to the cells, and the requirement for light delivery to the cells, which could limit future applications in deep tissue.

In conclusion, we have shown that by carefully tuning the experimental parameters, plasmonic cell fusion is capable of achieving extremely high fusion efficiencies in cancer cells. Specifically, at 7.5 × 10^10^ nanoparticles/ml and a 60 mJ/cm^2^ we have found that the high fusion efficiency is also accompanied by the generation of long-lived smaller cells. While the high sensitivity to the numerous experimental parameters may complicate the experimental design, it may allow flexibility in tuning the experimental protocol for very different applications, for example for obtaining specific fusion between an immune system cell and a malignant cell^42^, or for studying cancer metastasis following fusion between cancer and normal cells.

## Supplementary Information

Supplementary Video 1.

Supplementary Video 2.

Supplementary Information 1.

## Data Availability

The data that support the findings of this study are available from the corresponding author, upon reasonable request.
