# Supplementary material for: Optimization study of plasmonic cell fusion
Source: Sci Rep. 2022 May 3;12:7159. doi: 10.1038/s41598-022-11168-x (PMC9065096; doi:10.1038/s41598-022-11168-x)
Supplement: Supplementary file 3 — Supplementary Information 1. [file 41598_2022_11168_MOESM3_ESM.docx]

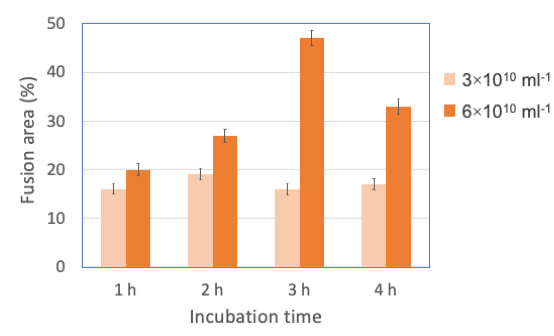


Supplementary Figure 1. Fusion vs incubation time 3 hours after irradiation. MDA breast cancer cells were incubated 1,2 3 and 4 hours before irradiation with two different concentration of gold nanoparticles: 3x10^10^ nps/ml (low concentration) and 6x10^10^ nps/ml (high concentration). The cells were irradiated by femtosecond laser beam by power 36 mJ cm^-2^ and 15 pulses.
